# Contagious Bovine and Caprine Pleuropneumonia: a research community’s recommendations for the development of better vaccines

**DOI:** 10.1038/s41541-020-00214-2

**Published:** 2020-07-24

**Authors:** Joerg Jores, Cynthia Baldwin, Alain Blanchard, Glenn F. Browning, Angie Colston, Volker Gerdts, Danny Goovaerts, Martin Heller, Nick Juleff, Fabien Labroussaa, Anne Liljander, Geoffrey Muuka, Vish Nene, Ran Nir-Paz, Flavio Sacchini, Artur Summerfield, François Thiaucourt, Hermann Unger, Sanjay Vashee, Xiumei Wang, Jeremy Salt

**Affiliations:** 1 Institute of Veterinary Bacteriology, University of Bern, Bern, Switzerland; 2 University of Massachusetts Amherst, Amherst, MA USA; 3 INRAE, Université de Bordeaux, UMR 1332 de Biologie du Fruit et Pathologie, 33882 Villenave d’Ornon, France; 4 Asia-Pacific Centre for Animal Health, Melbourne Veterinary School, University of Melbourne, Parkville, VIC 3010 Australia; 5 GALVmed, Doherty Building, Pentlands Science Park, Bush Loan, Penicuik, Edinburgh, EH26 0PZ Scotland UK; 6 Vaccine and Infectious Disease Organization-International Vaccine Centre, University of Saskatchewan, Saskatoon, SK Canada; 7 Friedrich-Loeffler-Institute - Federal Research Institute for Animal Health, Jena, Germany; 8 Bill & Melinda Gates Foundation, Seattle, WA USA; 9 International Livestock Research Institute, Nairobi, Kenya; 10 Central Veterinary Research Institute, Lusaka, Zambia; 11 Hadassah-Hebrew University Medical Center, Jerusalem, Israel; 12 Istituto Zooprofilattico Sperimentale Dell’Abruzzo e Del Molise G. Caporale, Teramo, Italy; 13 Institute of Virology and Immunology, Mittelhäusern, Switzerland; 14 Department of Infectious Diseases and Pathobiology, Vetsuisse Faculty, University of Bern, Bern, Switzerland; 15 Centre de coopération internationale en recherche agronomique pour le développement (CIRAD), Montpellier, France; 16 Animal Production and Health Section, Joint FAO/IAEA Division of Nuclear Techniques in Food and Agriculture, Vienna, Austria; 17 J. Craig Venter Institute, Rockville, MD USA; 18 Harbin Veterinary Research Institute, Chinese Academy of Agricultural Sciences (CAAS), Harbin, China

**Keywords:** Vaccines, Bacterial infection

## Abstract

Contagious bovine pleuropneumonia (CBPP) and contagious caprine pleuropneumonia (CCPP) are major infectious diseases of ruminants caused by mycoplasmas in Africa and Asia. In contrast with the limited pathology in the respiratory tract of humans infected with mycoplasmas, CBPP and CCPP are devastating diseases associated with high morbidity and mortality. Beyond their obvious impact on animal health, CBPP and CCPP negatively impact the livelihood and wellbeing of a substantial proportion of livestock-dependent people affecting their culture, economy, trade and nutrition. The causative agents of CBPP and CCPP are *Mycoplasma mycoides* subspecies *mycoides* and *Mycoplasma capricolum* subspecies *capripneumoniae*, respectively, which have been eradicated in most of the developed world. The current vaccines used for disease control consist of a live attenuated CBPP vaccine and a bacterin vaccine for CCPP, which were developed in the 1960s and 1980s, respectively. Both of these vaccines have many limitations, so better vaccines are urgently needed to improve disease control. In this article the research community prioritized biomedical research needs related to challenge models, rational vaccine design and protective immune responses. Therefore, we scrutinized the current vaccines as well as the challenge-, pathogenicity- and immunity models. We highlight research gaps and provide recommendations towards developing safer and more efficacious vaccines against CBPP and CCPP.

## Introduction

**Fig. 1:**
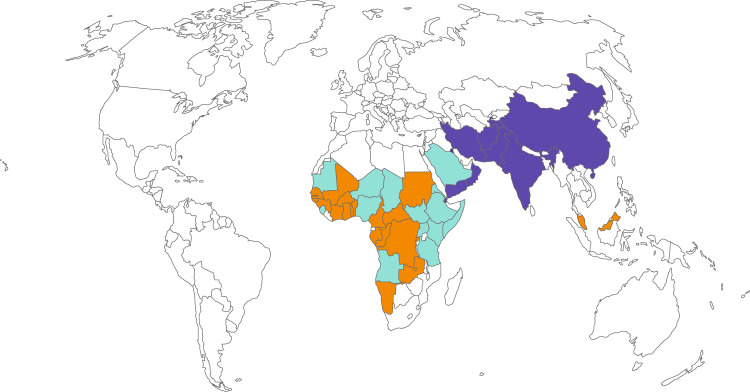
Occurrence of contagious bovine pleuropneumonia (CBPP) and contagious caprine pleuropneumonia (CCPP) from 2010 to 2019. Countries displayed in orange, purple, and turquoise reported confirmed cases of CBPP, CCPP, or both CBPP & CCPP, respectively. Data were collected from the website of the World Organization of Animal Health (www.oie.int).

Contagious bovine pleuropneumonia (CBPP) and contagious caprine pleuropneumonia (CCPP) are important transboundary diseases of cattle and goats especially in low and middle-income countries. Both diseases have multiple impacts. Pleuropneumonia is a very painful clinical state that is associated with reduced productivity, and, in its most dramatic outcome, death. Death rates are much higher for CCPP than for CBPP, however, increased mortality rates are associated with CBPP when infected cattle are introduced into naive herds^1^. The adverse affects on productivity include a reduction in milk production, daily weight gain, draft power, and fertility among others. Several surveys have ranked CBPP and CCPP constantly among the top five ruminant diseases for stakeholders across a range of livestock industry sectors. CBPP and CCPP are diseases that require precise diagnostic procedures in order to be detected. The current World Organization for Animal Health (OIE) prescribed diagnostic tests (cELISA, CFT) are fit-for-purpose at the herd level but are far from being optimal at the individual level. Given the passive surveillance approaches, limited resources, and limited diagnostic capacity in many parts of Africa and Asia, the current prevalence figures are probably an underestimate. Nevertheless, prevalence data retrieved from the OIE specific WAHID interface (Fig. 1 and Supplementary Figure) over the last 10 years suggests that there has not been any progress towards control of CBPP and CCPP, especially on the African continent. Control methods such as movement restrictions, quarantine, antibiotic treatment, and vaccination have had varying success. Reasons for failure to control the diseases are diverse covering social, economic, and political issues. Vaccination-related control strategies have been hampered by variable safety and efficacy of the vaccines and there is an urgent need for an improved vaccine for both diseases. New vaccines for these diseases must not only be safe and efficacious, but also cost-effective, scaleable and accessible to smallholder farmers and this should underpin rational vaccine design. Scientists from the research community reviewed the current knowledge on CBPP and CCPP as well as other mycoplasma discovery research projects to identify research gaps and agree recommendations at a workshop in Switzerland (Supplementary Notes). While control and eradication of these diseases would benefit from new, improved vaccines, there are many other social, economic and political research aspects that need to be addressed, but our focus in this article is the search for new and better vaccines.

## History of contagious bovine pleuropneumonia and contagious caprine pleuropneumonia and vaccines used for their control

The timing of the first report on an infectious disease is always difficult to verify in the absence of good diagnostic tools and modern high-speed communications that inform and shape our lives today. CBPP and CCPP were described in the relatively recent past with astonishingly precise descriptions of the clinical manifestations. The first records that mention CBPP originate from Bourgelat in France in 1765^2^, and then more notably from Albrecht von Haller in Switzerland in 1773^3^. Trade of animals from Europe to former colonies in ships, facilitated the entry of CBPP into the southern African continent in 1853^4^, then into Australia in 1858 and into North America in 1871. The causative agent, *Mycoplasma mycoides* subsp. *mycoides*, was the first mycoplasma ever cultivated in 1898^5^. These historical accounts have been confirmed and extended by recent molecular studies based on genomic data^6^, showing the existence of at least two independently imported lineages circulating in Africa^6^. After introduction and dispersal of CBPP, the disease was curbed by either detection and slaughter, combined with movement restriction (mainly executed by veterinary forces during the colonial period before 1960), or by the use of a combined live vaccine against Rinderpest virus and CBPP during the rinderpest eradication campaign^7^.

The first report of CCPP dates back to Algeria in 1873^8^. Subsequently, the disease spread to different countries in Europe, Asia, and Africa. For instance, a major outbreak struck South Africa after goats were imported from Turkey in 1881^9^. The fastidious mycoplasma that causes CCPP, namely *Mycoplasma capricolum* subsp. *capripneumoniae*, was only identified in 1976^10^. More recently, the Thrace region of Turkey experienced a CCPP outbreak with high mortality in 2002^11^ and the disease now seems to be endemic there^12^.

There are two vaccines being produced and used in the field for CBPP and CCPP. If manufactured and applied properly they have the capacity to prevent clinical disease and economic loss^13,14^. Interestingly, the CBPP vaccine is a live attenuated vaccine, while the CCPP vaccine is a bacterin.

The CBPP vaccine was attenuated by serial cultivation in eggs in the 1950’s and is based on the Tanzanian outbreak strain T1, with the 44th passage (T1/44) used as vaccine stock. The vaccine provides a level of protection that can be quantified by reduction in lung lesions in vaccinated and challenged cattle compared to unvaccinated and challenged animals. As it is a live vaccine, it can theoretically be combined with other live vaccines, an approach that was used successfully during the Rinderpest eradication campaign. This live bivalent vaccine containing streptomycin led to a marked reduction in CBPP in Rinderpest-affected countries^15^. Annual revaccination with the live vaccine is necessary to maintain protective immunity. It is relatively inexpensive and easy to produce at scale, as the cultivation of *M. mycoides* subsp. *mycoides* is much easier than cultivation of many other pathogenic *Mycoplasma* species, such as *M. hyopneumoniae* (the primary agent of enzootic pneumonia in pigs), *M. capricolum* subsp. *capripneumoniae* or *M. pneumoniae* (the cause of atypical pneumonia in humans). A minimal vaccine dose of 10^7^ colony-forming units of *M. mycoides* subsp. *mycoides* is recommended by OIE, so many doses can theoretically be achieved during commercial production from 1 ml of culture (which typically contains 10^9^ CFU/mL). In some instances, severe inflammation at the site of inoculation has been caused from this vaccine, especially after primary vaccination of cattle. This so-called Willems reaction is characterized by massive inflammatory reactions that can lead to skin sloughing at the vaccination site and in the worst case to death. Therefore, in the 1960s, Australian cattle were immunized at the tip of the tail with the V5 live vaccine, so that in the event of severe reactions the only effect was the loss of the tip of the tail. These reactions have an adverse effect on owner acceptance, and on animal welfare and marketing, leading to a low uptake of this vaccine. Anecdotal data suggest breed predispositions to these side effects. In addition, it has been shown that the T1/44 live vaccine strain is not fully attenuated and can cause disease when inoculated endobronchially^16^. Mass vaccination even with a moderate efficacy and duration of immunity vaccine (such as the current T1/44 live vaccine) alone is unlikely to eliminate CBPP according to an epidemiological model for CBPP transmission in pastoral herds of East Africa^17,18^. This includes a requirement for strict movement controls and the authors say it is validated by field observation. Furthermore, vaccine-derived immunity of at least 18 months is required to eliminate CBPP from individual herds according to recent study^13^. A combination of vaccination and antibiotic treatment of diseased cattle turned out to be most promising for CBPP control according to the epidemiological model^18^.

The current CCPP vaccine is a bacterin, and the OIE provides the guidelines for its production, which is basically culturing any field isolate and inactivating with saponin, which acts as an adjuvant. This vaccine was developed in the 1980s and 1990s^19,20^. The vaccine is administered as early as 4 months of age and revaccination is recommended every 6 months. The production of this vaccine is rather cumbersome given the fastidious growth requirements of the pathogen. Moreover, the vaccine dose of 150 µg total protein is relatively high, making the vaccine expensive compared to other caprine vaccines. The OIE protocol recommends 3 mg of the adjuvant saponin per vaccine dose, which exceeds standard saponin concentrations (0.3 mg per dose) for small ruminants. The main drawback of this otherwise safe vaccine is the inflammatory reactions at the site of vaccination^21^, possibly due to the saponin adjuvant, and the short duration of immunity of only 6 months to maximally 1 year. These limitations might be overcome with new adjuvant formulations. Given the envisaged eradication of Peste des Petits Ruminants (PPR), a live CCPP vaccine that can be combined with the PPRV vaccine and other live vaccines would be desirable. Such a vaccine would save cost in terms of production and logistics associated with vaccine delivery. Given the widespread use of the live T1/44-based CBPP vaccine despite its residual pathogenicity^16^, live strains of *M. capricolum* subsp. *capripneumoniae*, which can be safely injected subcutaneously, should be tested for their ability to induce a protective immune response.

The limitations of the current CBPP and CCPP vaccines have led to a search for better vaccines. A lapinized vaccine based on the passaged strain Ben-1 has been used during the eradication of CBPP in China in the framework of a comprehensive eradication program^22^. This vaccine is reported to provide 95–100% protection over 2 years, which is superior to other mycoplasma vaccines and most commercial ruminant vaccines. However the method used for its production, intraperitoneal inoculation of sheep, is unacceptable under current animal welfare standards.

An experimental vaccine batch based on the lapinized strain but produced in culture media was compared with the commercial T1/44 vaccine in an in vivo trial on African cattle breeds, but its protective capacity was not superior to the commercial T1/44 (https://www.galvmed.org/galvmedat10/05-key-achievements.html). One of the key learnings of eradication of CBPP in China is that the lapinized and its sheep-adapted vaccine were paramount for the establishment of immunity zones around enzootic regions besides the other undoubtedly important control measures such as movement control as well as detection and slaughter.

Recently, using a reverse vaccinology approach researchers identified several candidate vaccine antigens that conferred protective immune responses to experimental CBPP challenge^23^. Further work has been ongoing to optimize the vaccine (scale up, duration of immunity, field testing, and comparison with T1/44 vaccine) with commercial acceptance in mind.

Any new vaccine should be tested under different field conditions and different control strategies identified using epidemiological models and previous local experience if available, to guide and inform national and regional control policy makers. An additional consideration is the uptake of livestock vaccines by smallholder farmers and marginalized populations which may require different strategies depending on factors such as the type of disease to be tackled, the value of the livestock species, the market size, profitability as well as the support of national and regional vaccine manufacturers among others. Vaccine adoption is influenced by availability of, access to and demand for vaccines. Global and national vaccine campaigns differ from market-driven approaches. The different strategies to increase adoption of animal vaccines by smallholder farmers especially in low and middle-income countries have been very capably reviewed recently^24^ and, as mentioned for implementation of control strategies, will not be further discussed.

## Virulence traits, pathogenicity models, and potential vaccine targets for CBPP and CCPP

Most work on deciphering the factors that drive pathogenicity in these two pathogens has been performed on CBPP, probably because of European outbreaks in the 1980s and 1990s, which subsequently attracted research funding to several European laboratories. It has been proposed that African and European strains differ in virulence, this was based on an in vivo in-contact challenge study employing 2 groups of 2 animals each, which were infected with different *M. mycoides* subsp. *mycoides* strains. Strictly speaking only 3 animals were used, since one animals stayed seronegative after the first in-contact challenge and was subsequently reused in the other challenge group. Clinical signs of all 4 in-contact animals were very mild (nasal discharge) and pathomorphological changes were not seen in any of these in-contact challenged animals irrespective of the strain used^25^. The only difference observed was an earlier onset of seroconversion in animals in-contact with a donor infected with the African strain Afadé^26^. Later on it was shown by others that experimentally infected cattle developed different seroconversion profiles, described as early high responders, late high responders, and low responders^27^.

The African and European strains differed in a genetic locus encoding the ATP-binding cassette transporter proteins GtsA, GtsB, and GtsC, which were proposed to be involved in glycerol transport. The European strains missing this locus produced lower concentrations of H_2_O_2_^28^. Based on the association between this metabolic difference and the late seroconversion after infection with the European strain L2, it was concluded that H_2_O_2_ production is a virulence trait. A mycoplasma metabolic enzyme called glycerol phosphate oxidase (GlpO) was identified as central in the production of peroxide in the presence of physiological glycerol^29^. This mechanism has been proposed as the main virulence mechanism of CBPP^30^ without confirmation using ex vivo or in vivo data according to Falkow’s postulates^31^. More recently, immunization experiments using recombinant GlpO followed by challenge did not demonstrate protection, which may be because either H_2_O_2_ production is not a main virulence trait or that GlpO itself is mainly cytoplasmic^32^, preventing antibodies from inhibiting its function.

Several other virulence traits have been suggested, but mainly based on in vitro systems with little or no data available from ex vivo or in vivo models. Since successful challenge of cattle with *M. mycoides* subsp. *mycoides* is difficult (see below) and efficient genetic tools to produce isogenic mutants are unavailable^33^, researchers have focussed instead on the caprine pathogen *M. mycoides* subsp. *capri*, in order to decipher virulence traits in *Mycoplasma mycoides* and test them in vivo. These studies have used synthetic genomics techniques, which allow unprecedented precise genome engineering of mycoplasmas^34–39^ and a caprine challenge model^40–42^. These experiments have provided in vivo evidence that capsular polysaccharide is a virulence trait in *Mycoplasma mycoides*^40^, as suggested more than 40 years ago^43^. They also have enabled the generation of a temperature-sensitive mutant of *M. mycoides* subsp. *capri* by targeting the essential gene *obg*^42^. However, this mutant did not seem to induce a strong protective immune response against a challenge with the wild type strain.

Another candidate virulence factor is the MIB-MIP system that is proposed to degrade immunoglobulins^44^. It is not difficult to imagine an extracellular pathogen (the current understanding of the habitat of these pathogens) would be shielded by interference with the function of host immunoglobulins, thus contributing to survival and pathogenicity. The in vitro proteolytic activity of the system has recently been confirmed in vivo^41^.

Last but not least, lipoproteins and variable surface proteins are candidate virulence factors that definitely deserve more research^45^. Like the capsular polysaccharide and the MIB-MIP system these cellular components are located on the pathogen’s surface and are therefore the primary molecules interacting with the host. It is known that for certain *Mycoplasma* species particular lipoproteins act as variable surface antigens [for review see:^46^] and facilitate evasion of the host’s immune response. Some of the lipoproteins show some degree of amino acid sequence identity across different *Mycoplasma* species, but their exact role in virulence has not been deciphered for *M. mycoides* subsp. *mycoides* and *M. capricolum* subsp. *capripneumoniae*. It has been suggested that they contribute to an overwhelming immune response that leads to immunopathological consequences^47^. The lipid moieties of *Mycoplasma pneumoniae* lipoproteins have been recently suggested as causative factor of vaccine-enhanced disease using a mouse model^48^.

## Research gaps to be addressed

**Fig. 2:**
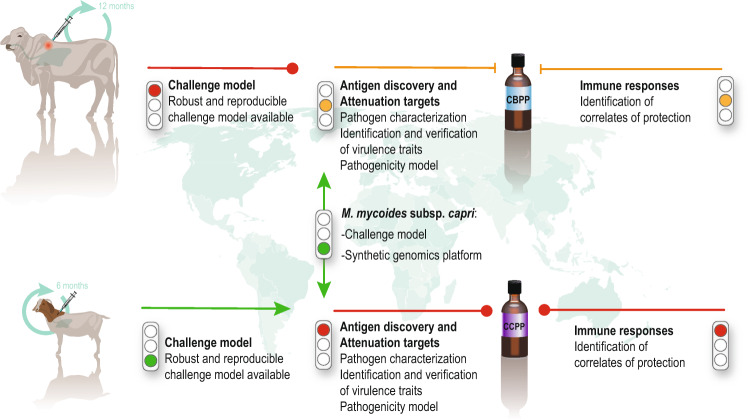
Cartoon displaying the major research blocks that influence rational vaccine design. The characteristics of the current CBPP and CCPP vaccines are displayed on the left. The state of knowledge is characterized by traffic lights (red—missing; yellow—partly available; green—available).

For clarity and a more systematic approach we have subdivided this important area into three topics. We summarized the current state of research blocks (Fig. 2) and a priority list of recommended research needs (Table 1).

**Table 1:** Five top research priorities for the development of CBPP and CCPP vaccines.

Priority	Contagious Bovine Pleuropneumonia	Contagious Caprine Pleuropneumonia
1	Development of a robust challenge model	Characterization of surface-localized virulence factors
2	Ex vivo and in vivo verification of surface-localized virulence factors using harmonized caprine models and *M. mycoides* subsp. *capri* (see Table 2)	Generate mutants and confirm attenuation in harmonized caprine ex vivo models
3	Generate *M. mycoides* subsp. *mycoides* mutants based on mutations that led to attenuation in an *M. mycoides* subsp. *capri* model (see priority 2) and test them as a live vaccine in vivo	Test candidate mutants for attenuation and induction of immune responses in vivo
4	Extend and revisit immunological knowledge based on correlates of protection using the novel challenge model (see Priority 1): characterize the innate and adaptive immune responses (local and systemic) after infection of vaccinated and naïve animals	Define immunological correlates of protection: (1) perform adoptive transfer of caprine IgG harvested from immune animals following immunization & challenge; (2) characterize the innate and adaptive immune responses (local and systemic) after infection of vaccinated and naïve animals
5	Characterize the Willems reaction and the mycoplasma factors that drive it	Applying systems immunology to improve the adjuvant formulation used for the current bacterin vaccine

### Challenge models

A reproducible challenge model is a *conditio sine qua non* for the development of a vaccine. Most mycoplasmas including *M. mycoides* subsp. *mycoides* and *M. capricolum* subsp. *capripneumoniae* are host-target tissue specific. We should focus on the natural host and improve, if needed, the available challenge models. But which challenge models are available? For CCPP, of late a novel challenge model has recently been reported^49^, based on the recent Kenyan outbreak strain ILRI181^50^. This model is based on a combination of two subsequent inoculations of aerosols of cultures into the nasal cavity of goats followed by a trans-tracheal inoculation. Morbidity of 100% and a mortality of 50–60% was seen in infected animals, closely mimicking natural infection^49^. Improvements of the challenge model are possible, including titration of the challenge dose, use of an aerosol chamber for infection as has been used for experimental infections with other *Mycoplasma* species^51,52^, and whether either the aerosol or the trans-tracheal inoculations are dispensable to allow simplification of the procedure. However, given the robustness of the model thus far and its potential to be implemented in resource-poor settings, improvement of the model is currently a lower priority (Fig. 2). We encourage the development and employment of caprine airway epithelial cell cultures such as used in the human *Mycoplasma* field^53–56^.

The current challenge models for CBPP are less advanced and are suboptimal, as they have resulted in a variety of outcomes ranging from no clinical disease^26^ to a wide spectrum of pathological lesions^23,47,57–59^. A common feature of these models is the intratracheal inoculation of a *Mycoplasma* culture using a tube that is inserted via the nasal or oral cavity while the animal is standing or in lateral recumbency. In the past, fresh cultures were used for inoculation, however, it has now been demonstrated that frozen stock cultures work well for both pathogens^60,49^. The instillation of a solution of agar after inoculation of the mycoplasma broth culture has been show to be dispensable^60^. Trans-tracheal inoculation is easy to perform in *Bos taurus* cattle but is difficult in *Bos indicus* animals, as the dewlap in these animals impedes access and a Willems reaction is likely to occur. Given the range of disease severity with intratracheal inoculation reported so far, systematic development of a robust and reproducible challenge model for CBPP is clearly a priority (Fig. 1, Table 1). It needs to be based on a low passage field isolate, using cattle over 1 year of age and a defined dose of the organism. A good option would be to try aerosolized cultures and repeated inoculations, as this best mimics the natural route of infection. As a starting point, results obtained with *M. bovis* using an aerosol chamber to induce experimental infection in calves should provide some guidelines for further development^51^. A standardized challenge model, preferably using a single aliquoted frozen challenge stock, would allow data to be exchanged and compared more easily, a critical requirement given the high costs and logistical challenges associated with large animal trials. This way, the research community could benefit the most from data generated in in vivo trials, which generally trump data derived from in vitro and ex vivo assays.

In-contact infection models were not considered a practicable alternative as the timing of individual infection and disease signs are difficult to synchronize because of differences of interactions between animals, which influence transmission of disease. Therefore, such models are intrinsically more variable, require a large number of animals and are not the best option for the read out of immunological parameters over time. Moreover, recently established ex vivo models^61,62^ for deciphering pathogenicity mechanisms and identifying virulence traits may prove beneficial (Table 1).

## Candidate vaccine antigens and attenuation targets - filling the knowledge gap on Mycoplasma

Mycoplasmas lack a cell wall and due to their inherent minute size one might assume that they are easily characterized, but this is not the case. In fact, we know relatively little about the structure and physiology of *M. mycoides* subsp. *mycoides* and *M. capricolum* subsp. *capripneumoniae*. This is influenced by the classification as BSL3 pathogens in some countries, restricting many research laboratories from working with them, and by the lack of genetic tools to induce defined mutations. However, the advent of synthetic genomics has paved the way for mutagenesis of a closely related subspecies of *M. mycoides* subsp. *mycoides* namely *M. mycoides* subsp*. capri*^35–38^. The availability of diverse next generation sequencing platforms has enabled researchers to characterize the genomes of *M. mycoides* subsp. *mycoides*^63^ and *M. capricolum* subsp. *capripneumoniae*^50,64^, demonstrating relatively little sequence diversity^6,65^.

What we lack at present is characterization of the pan and core genome of *M. mycoides* subsp. *mycoides*, *M. capricolum* subsp. *capripneumoniae* and the entire “*M. mycoides* cluster”. This information is likely to help us identify common genes among mycoplasmas that play a role in fitness and persistence in hosts and to identify new vaccine targets or sites for attenuation. A detailed transcriptomics analysis, preferably combined with proteomics studies is also missing. This would provide baseline data that could be used to tease apart operons and promoters and enable us to better understand the organisms’ physiological pathways as well as improve the annotation of the genomes for the sake of rationale vaccine design.

The mycoplasma metabolism depends on scavenging a wide range of host molecules, as mycoplasmas are unable to synthesize many of the precursors for their macromolecules (including proteins, nucleic acids, and lipids). This scavenging lifestyle requires transporters that span the cell membrane and translocate the scavenged molecules to the enzymatic apparatus inside the mycoplasma cell. Interference with such transporters is likely to affect the growth and viability of mycoplasmas substantially, so essential transporters are likely to be promising targets in future rationale vaccine efforts. Candidates have already been identified in other *Mycoplasma* species that are present in the “*Mycoplasma mycoides* cluster”, including the putative oligopeptide/dipeptide (opp/dpp) ATP-binding cassette (ABC) transporter^66,67^. Therefore, a combination of in silico analysis and OMICS data (proteomics, transcriptomics, metabolomics) is likely to enable us to identify transporters that should be target for future characterization.

However, we need to know more about lipoproteins and other surface-exposed proteins of these mycoplasmas, and particulary their function and abundance in order to identify protective antigens to be tested later on (Table 1). The core in vitro surface proteome of *M. mycoides* subsp. *mycoides* has been suggested to include about 50 lipoproteins and other membrane proteins^68^. Some of these have been shown to be immunogenic^69^ and even to induce a protective immune response^23^, while others have been suggested to contribute to immunopathology^47^. Understanding of the surface proteome of *M. capricolum* subsp. *capripneumoniae* is lagging behind. Many genes encoding putative lipoproteins and transmembrane proteins have been identified *in silico*^50,64,70^, but data regarding their expression, function and immunogenicity are lacking. It will be important to first confirm the cellular localization of these proteins in *M. mycoides* and *M. capricolum* using either available fluorescent molecules (e.g., mCherry, mNeon)^71^ or tags coupled with electron microscopy among others. This work would allow the research community to build a list of surface-exposed virulence candidates that can be further tested in ex vivo and in vivo systems discussed above. In an effort to initiate such a process, we have listed the candidate virulence traits that we believe require immediate attention (Table 2).

**Table 2:** Prioritization of candidate virulence factors for investigation according to Falkow’s postulates.

Priority	Candidate virulence factor	Comment
1	L-alpha-glycerophosphate oxidase (GlpO)	Knock out mutants are available for *M. mycoides* subsp. *capri*, the role of this candidate virulence factor requires immediate investigation, as it is dispensable for virulence in *M. gallisepticum*
2	MIB-MIP system	Knock out mutants are already available for *M. mycoides* subsp. *capri*, in vivo activity has been proven and this is a top candidate virulence factor
3	Different DUF groups of lipoproteins	Individual lipoproteins and pathogen specific DUF groups of lipoproteins need to be tested for their role in virulence
4	Variable surface proteins	Have been shown in vitro and in vivo to be functional in mycoplasmas
5	Oligopeptide/dipeptide (opp/dpp) ATP-binding cassette (ABC) transporter	Has been shown in *M. agalactiae* to be a candidate virulence factor
6	6-phospho-beta-glucosidase (Bgl)	Correlations indicate a role in cytotoxicity

Some of the mycoplasma lipoproteins identified so far can be classified into different families, as they possess conserved domains of unknown functions (DUFs). Some of these families are found throughout the phylum *Firmicutes* (which includes the class *Mollicutes*) while others appear to be “mycoplasma-specific”. Functional studies of these proteins are not trivial as functional redundancy is likely to occur between the different family members. Until efficient genetic tools are available for *M. mycoides* subsp. *mycoides* and *M. capricolum* subsp. *capripneumoniae*, we suggest using the closely related pathogens *M. mycoides* subsp. *capri* and *M. capricolum* subsp. *capricolum*, respectively, as a model to characterize these lipoproteins (Fig. 2). The capacity to delete multiple genes or even subgenomic fragments in the genomes of *M. mycoides* subsp. *capri* and *M. capricolum* subsp. *capricolum* will allow the generation of strains in which all members of a DUF family have been removed. Using such a mutagenesis system, five genomic regions of *M. mycoides* subsp. *capri* strain GM12 comprising 68 genes were deleted recently^41^. In addition to the glycerol metabolism-related genes, the deleted genes encoded 18 lipoproteins and 21 transmembrane proteins. This resulted in the complete attenuation of this otherwise pathogenic strain. The same synthetic genomics tools have been used to graft heterologous subgenomic *Mycoplasma* fragments into *M. mycoides* subsp. *capri* (unpublished data), generating a useful tool for future characterization of a subset of *M. mycoides* subsp. *mycoides* and *M. capricolum* subsp. *capripneumoniae* target molecules.

Another interesting feature of mycoplasmas is their exosecretion of bacterial compounds that can interact with their hosts. *M. mycoides* cell membranes have been shown to have several protrusions that can themselves have several constrictions^63^. Extracellular vesicles have also been shown to be released by mycoplasmas^72^. Other mycoplasmas, including *M. hyopneumoniae*, shed extracellular domains of proteins^73^ and this has also been observed for *M. capricolum* subsp. *capripneumoniae* and *M. mycoides* subsp. *mycoides*^74^. The role of these secreted vesicles and proteins in the pathogenesis of mycoplasmoses, if any, needs to be determined in future studies.

In the interest of maximizing the benefit obtained from limited resources we suggest focusing research activities on a defined set of mycoplasma strains in order to generate synergies and facilitate comparisons between in vivo, ex vivo, and in vitro data (Table 1).

## Defining correlates of immunity and characterizing immune responses after infection

Most of the immunological data generated this far has been collected by the research groups at CIRAD, the International Livestock Research Institute and the Kenya Agricultural & Livestock Research Organization, who have been undertaking vaccine development work. In the interest of time and space we do not include in vivo data that have been collected from rodent models, which are not likely to be as informative about protective immune responses in domestic ruminants. In their natural hosts, immunizations with a live CBPP vaccine or a CCPP bacterin induce a protective immune response. This immunity needs to be dissected to foster rational design of improved vaccines (Table 1).

We suggest performing adoptive transfer experiments with purified antibodies from convalescent, diseased, and healthy animals to animals that are subsequently infected experimentally. Such activities should be initiated with CCPP, as the infection model is well established, and large amounts of sera have been collected from convalescent animals at necropsy during a recent trial^49^. If the adoptively transferred antibodies are protective, we can focus on characterization of the spectrum of specificities of the antibodies using bead-based assays and bioinformatic network analysis of B-cell responses in protected animals. A similar experiment should be done with CBPP, when a better challenge model is available.

Traditional methods of determining the protective role of T-cell mediated immunity, by depleting specific subsets of T cells have been performed for CBPP^57^. Such depletion experiments require large quantities of monoclonal antibodies directed against markers on ruminant T cells, and often necessitate repeated administration. Their interpretation can be misleading because T cells are a component of an interactive system and removing any one element can have an impact on the whole system. Furthermore, many cell surface markers are not unique to one population. Therefore, alternative approaches may be useful, such as those arising from the field of systems immunology^75^ and systems vaccinology^76^, which have recently been adapted from humans to livestock species^77,78^. High-throughput technologies such as RNA sequencing coupled with data analysis allow the interrogation of hosts’ intracellular and intercellular interactions to understand the entire immune system better. It permits exploration of mechanistic insights, which enables vaccines to induce protective immune responses. The principle of such analyses is the collection of very large data sets by examining responses of immune cells using advanced immunological techniques and transcriptomic analyses. Changes in the transcriptional modules and changes in the immune responses can be analysed for any correlations to identify elements of the innate and adaptive immune response that promote protection in ruminants. It can be used to analyse both live and inactivated vaccine responses and correlate them with protection and to identify a suitable adjuvant that promotes longlasting immunity^76^. Applying such technologies to the current bacterin CCPP vaccine and novel CBPP and CCPP vaccine candidates will inform on the mechanism of protection, including both antibody responses and responses by different types of T cells, including the role of T helper (Th)1, Th2, and Th17 or regulatory T cells (Table 1). Although these approaches are resource intensive and require bioinformatic and immunological expertise, they will enable a more rational design of vaccine and accelerate the development of improved vaccines. Future research focusing on components of mucosal immunity such as secretory IgA levels that can prevent infection is also required, as it is known with other mycoplasma vaccines that systemic antibody does not necessarily correlate with protection against lung pathology^79^.

The characterization of the innate immune response is also paramount, as the innate immune response is likely to determine the outcome of infection or immunization by directing the type of adaptive immune response that ensues, and is also the first line of defence against the pathogen. It is self-evident that granulocytes^80^ and macrophages^81^ infiltrating the site of infection shape the outcome of disease and the pathogenicity. Therefore, their interaction with the pathogen and its components needs to be closely followed to generate a more complete picture of the mechanisms underlying the development of lesions initiated by either the pathogen or the host immune response. New and improved adjuvants and immune modulators are available that can increase the duration of immunity and direct the immune response towards more protective responses. These should be evaluated, using the data generated in the experiments described above to guide selection of the most appropriate candidate vaccines.

## Prioritization of research efforts towards a novel prophylactic vaccine

Prioritization largely depends on the point of view of individual researchers, with microbiologists favouring characterization of the pathogen and immunologists favouring the characterization of the immune responses. However, a closer interaction between these two groups is needed to best tackle pathogens that kill our livestock in such large numbers. After rigorous scientific discussions, our suggested priority actions are shown in Table 1. We believe these approaches will guide development of better vaccines that can be used as tools in the control of these two important pathogens.

However, even the most efficacious vaccine does not alone imply its adoption and successful disease control. The Rinderpest eradication taught us that any vaccination campaign needs to involve many stakeholders such as epidemiologists and social scientists to develop and implement policies and strategies to achieve successful disease control^7^.

## Supplementary information

Supplementary Information

## Data Availability

All relevant data are included in the manuscript and Supplementary Information.
