# Supplementary material for: Contagious Bovine and Caprine Pleuropneumonia: a research community’s recommendations for the development of better vaccines
Source: NPJ Vaccines. 2020 Jul 24;5:66. doi: 10.1038/s41541-020-00214-2 (PMC7381681; doi:10.1038/s41541-020-00214-2)
Supplement: Supplementary file 1 — Supplementary Information [file 41541_2020_214_MOESM1_ESM.pdf]

Jores et al., 2020; Contagious Bovine and Caprine Pleuropneumonia: a research community's recommendations for the development of better vaccines

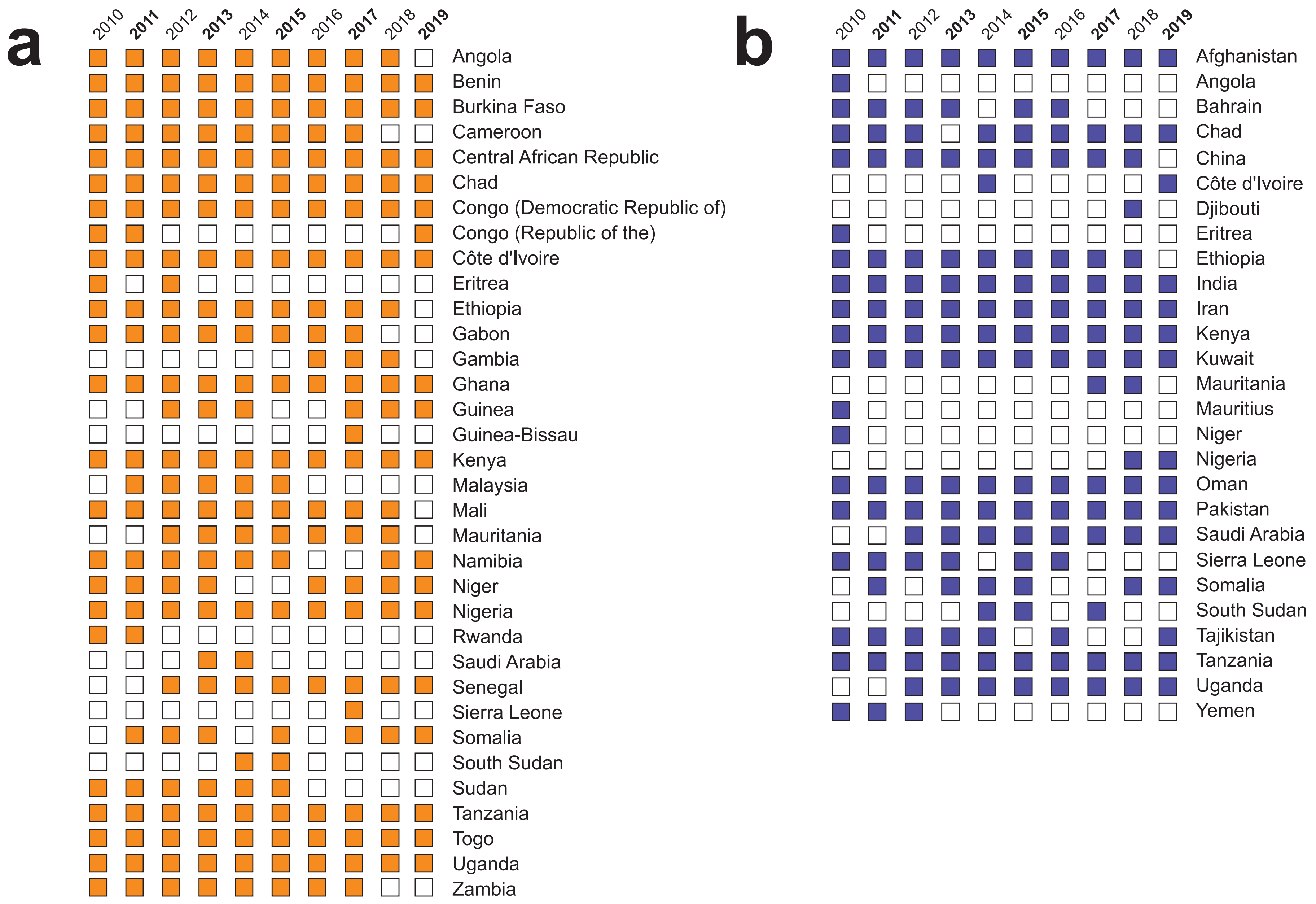

Supplementary figure: Ten year record of confirmed CBPP (a) and CCPP (b) based on OIE data

Supplementary Notes: Program information of the workshop entitled 'Contagious bovine and caprine pleuropneumonia, an update on the current knowledge base'
Venue: House of the University of Bern, Switzerland

January 31, 2018

09:00 Overview of the workshop and logistics (J. Salt & J. Jores)

Contagious bovine pleuropneumonia (CBPP): Part I (chaired by F. Labroussaa)

09:15 Overview of CBPP incl. disease, epidemiology and control strategies (F. Thiaucourt)
09:55 CBPP (and CCPP) vaccine production and quality control in Africa (H. Unger)
10:15 Diagnostic assays (M. Heller)
11:00 Interlude: Rational and empirical vaccine development in veterinary medicine (C. Baldwin)
11:30 Efficacy study of the BEN-1-based life vaccine in Africa (X. Wang)

Contagious bovine pleuropneumonia (CBPP): Part II (chaired by A. Liljander)

13:30 Development of a subunit vaccine (V. Gerdts)
13:55 Antimicrobial efficacy studies carried out in Kenya and Zambia (G. Muuka)
14:20 Synthetic biology efforts towards a live vaccine (S. Vashee / A. Blanchard)
14:45 Towards a better challenge model for CBPP (Vish Nene)
15:20 Small group discussions on research gaps (4 groups, bus stop)
16:40 Summaries of research gaps (C. Baldwin, G. Browning, A. Colston, D. Gooverdts)
17:10 Interlude: Development of vaccines against avian mycoplasmas (G. Browning)

February 1, 2020

Contagious caprine pleuropneumonia (CCPP): Part I (chaired by G. Muuka)

09:00 Overview of CCPP incl. disease, epidemiology and control strategies (A. Liljander)
09:40 Diagnostic assays available for CCPP (H. Wesonga)
09:55 Set up of a challenge model for CCPP (F. Sacchini)
10:20 Development of a live attenuated vaccine for CCPP (J. Jores)
11:05 Rationale design of a vaccine chassis delivering *Mccp* antigens (F. Labroussaa)
11:30 Interlude: Systems immunological approaches for vaccine improvement (A. Summerfield)

Contagious caprine pleuropneumonia (CCPP): Part II (chaired by A. Colston)

13:30 Small group discussions on research gaps (4 groups, bus stop)
14:50 Summaries of research gaps (C. Baldwin, G. Browning, A. Colston, D. Gooverdts)
15:40 Interlude: Development of a vaccine against human *M. pneumoniae* infections (N. Ran-Paz)

Needs and future prospects for CBPP and CCPP disease control

16:10 BMGF & GALVmed activities in the field of veterinary *Mycoplasma* (N. Juleff & J. Salt)
16:25 Open discussion: How to foster CBPP and CCPP vaccine development? (J. Jores)
17:10 Wrap up session (J. Salt & J. Jores)

Jores et al., 2020; Contagious Bovine and Caprine Pleuropneumonia: a research community's recommendations for the development of better vaccines

List of workshop participants:

Name	Affiliation
Prof Cynthia Baldwin	UNIVERSITY OF MASSACHUSETTS AMHERST, USA
Prof Alain Blanchard	INRAE, FRANCE
Prof Glenn Browning	UNIVERSITY OF MELBOURNE, AUSTRALIA
Dr Angie Colston	GALVMED, KENYA
Prof emeritus Joachim Frey	UNIVERSITY OF BERN, SWITZERLAND
Prof Volker Gerdts	VIDO, CANADA
Dr Danny Gooverdts	CONSULTANT, BELGIUM
Dr Andy Hall-Ponselè	BIOTANGENTS LIMITED, UK
Dr Martin Heller	FLI, GERMANY
Prof Joerg Jores	UNIVERSITY OF BERN, SWITZERLAND
Dr Nick Juleff	BILL AND MELINDA GATES FOUNDATION, USA
Dr Fabien Labroussaa	UNIVERSITY OF BERN, SWITZERLAND
Dr Anne Liljander	ILRI, KENYA
Dr Elharrak Mehdi	MCI, TUNESIA
Dr Geoffrey Muuka	CVRI, ZAMBIA
Dr Vish Nene	ILRI, KENYA
Prof Ran Nir-Paz	HADASSAH-HEBREW UNIVERSITY, ISRAEL
Dr. Flavio Sacchini	IZS, ITALY
Dr Jeremy Salt	GALVMED, UK
Prof Artur Summerfield	INSTITUTE OF VIROLOGY AND IMMUNOLOGY, SWITZERLAND
Dr François Thiaucourt	CIRAD, FRANCE
Dr Hermann Unger	JOINT FAO/IAEA PROGRAM, AUSTRIA
Prof Sanjay Vashee	JOHN CRAIG VENTER INSTITUTE, USA
Dr Xiumei Wang	CAAS, HARBIN, CHINA
Dr Hezron Wesonga	KALRO, KENYA
